# Safety and efficacy of a modified WALANT technique using undiluted adrenaline during open surgical carpal tunnel release: a prospective report of 308 procedures

**DOI:** 10.1186/s13018-023-04369-1

**Published:** 2023-11-17

**Authors:** Mohamed Mostafa Kotb, Usama Farghaly Omar, Ahmed A. Khalifa

**Affiliations:** 1 Upper Limb and Reconstructive Microsurgery Unit, Orthopedic Department, Assiut University Hospital, Asyût, Egypt; 2 Orthopaedic Surgery Department, Al-Hekma Specialized Hospital, Asyût, Egypt; 3 Orthopedic Department, Qena Faculty of Medicine and University Hospital, South Valley University, Kilo 6 Qena-Safaga Highway, Qena, Egypt

**Keywords:** Carpal tunnel syndrome, WALANT, Wide awake, Epinephrine

## Abstract

**Purpose:**

The current study aimed to report on the safety and efficacy of utilizing a modified WALANT (mWALANT) technique during open surgical carpal tunnel release (CTR), where we used undiluted epinephrine compared to the originally described WALANT technique.

**Methods:**

From January 2015 till the end of June 2021, 200 patients (175 (87.5%) were females) who presented with carpal tunnel syndrome, either bilateral (108 (54%) patients) or unilateral (92 (46%)) were included, formulating a total of 308 procedures. Open surgical CTR was performed as a daycare procedure by the same surgeon. The mWALANT injectable mixture was prepared by mixing 8 CC of 2% lidocaine HCl + 1 CC of 0.25 mg/1 ml epinephrine without dilution (2.5 times the concentration used in the original WALANT technique). The injection was performed before draping.

**Results:**

The patients’ average age at surgery was 42.88 ± 13.03 years old; they were followed up for an average of 31 ± 17.17 months. The average operative time was 9.5 ± 1.87 min. None (0.0%) of the patients needed top-up of local anesthesia or shift into general anesthesia, and no (0.0%) patients needed postoperative hospital stay. The average VAS during the surgical procedure was 2.5 ± 2.1, mainly reported during infiltration of the local anesthesia; no patients reported discomfort during the surgical procedure itself. 180 (90%) patients reported a full return to their usual preoperative ADL after an average of 4.7 ± 1.2 weeks. No (0.0%) postoperative fingers ischemic or temperature changes. Two (1%) patients experienced an adrenaline rush in the form of tachycardia that needed sedation and close monitoring by the anesthesiologist; they were discharged on the same day. One (0.5%) patient (who had uncontrolled diabetes mellitus) showed a superficial wound infection which resolved after conservative management.

**Conclusions:**

Using undiluted epinephrine during the mWALANT technique is safe and effective. There is no need to wait until the drugs fully function, and no epinephrine-related complications were encountered apart from occasional adrenaline rush symptoms.

## Introduction

Median nerve compression neuropathy at the wrist level, commonly known as carpal tunnel syndrome (CTS), is a common problem affecting the patient’s activities of daily living (ADL) [1, 2]. Its management entails various lines, including conservative options (medications, night splints, local steroid injection, and physiotherapy) [2, 3], which, if failed, surgical decompression could be performed either open or endoscopic [4–6].

Herbert Galloway reported the first carpal tunnel release (CTR) by surgical release in 1924 [7]; since then, surgical decompression has become the standard management line if conservative lines fail [6]. This procedure could be performed under various anesthesia techniques, including general, regional, intravenous regional, or even local anesthesia [6, 8]. However, in an attempt to decrease the waiting lists and save costs, a Canadian hand surgeon, Dr. Lalonde, introduced the wide-awake local anesthesia no tourniquet (WALANT), which is a technique in which a local anesthetic accompanied by vasoconstrictive agents are used, which eliminates the need for sedation or tourniquet [9–12].

In the original WALANT technique description, injection of 1:100,000 epinephrine locally at the surgical site (acting as a hemostatic agent) and waiting for 30 min (average 26 min) for maximum vasoconstriction effect [10, 13, 14]. However, some of the shortcomings of this technique are related to adjusting the exact mixture solution and waiting time for the onset of action of vasoconstriction; furthermore, the technique of injection and specific needle size and slow injection that some surgeons prefer to avoid following [15].

The current study aimed to report on the safety and efficacy of utilizing a modified WALANT (mWALANT) technique during open surgical CTR, where we used undiluted epinephrine compared to the originally described WALANT technique.

## Patient and methods

The current study was approved by our institution’s Institutional Review Board Committee. All participating patients signed informed consent after discussing the study protocol and possible complications.

From January 2015 till the end of June 2021, we included patients presented with CTS (unilateral or bilateral) refractory to conservative lines of management willing to undergo surgical decompression and to approve their participation in the study. Patients presented with recurrent CTS after previous surgical decompression or patients refusing to participate in the study were excluded. This resulted in the inclusion of 200 patients during the study period, and 175 (87.5%) of them were females.

Preoperative evaluation and confirmation of the diagnosis were performed through clinical examination, including positive Phalen’s and Tinel’s signs, assessing the motor power of abductor pollicis brevis (APB). Furthermore, nerve conduction studies were performed to confirm the diagnosis. A cervical X-ray was obtained for all patients to exclude the possibility of cervical radiculopathy.

### Surgical technique

The patient was operated upon in a supine position while a side table supported the arm and hand. In all procedures, an anesthesiologist monitored patient vitals during the injection process and throughout the procedure. Furthermore, the anesthesiologist was prepared to interfere if the local anesthesia failed or in case of undue complications related to the injected drugs.

**Fig. 1:**
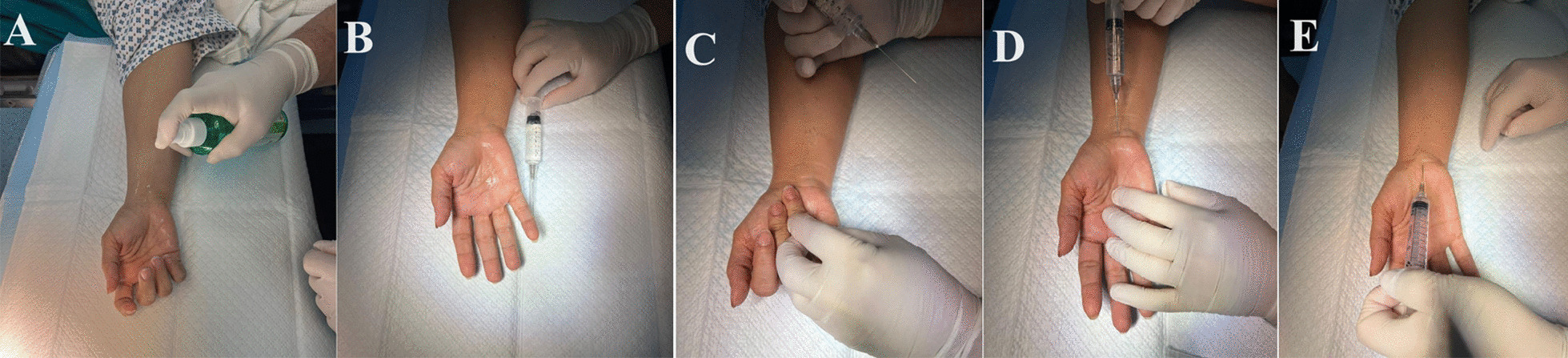
Injection technique using mWALANT technique, **A** sterilization of the area to be injected, **B** the mixture to be injected, **C** determining the appropriate level of the surgical incision for optimum injection of the mixture, **D** injection distal to the wrist crease, **E** injecting proximal to the wrist crease

*Local anesthesia (mWALANT) technique:* this was performed first before draping. The mWALANT injectable mixture was prepared by mixing 8 CC of 2% lidocaine HCl + 1 CC of 0.25 mg/1 ml epinephrine without dilution, which is 2.5 times the concentration used in the original WALANT technique [12]. The mixture was injected using a 22-gauge needle and locally infiltrated into the affected hand without prior intravenous sedation. Infiltration included the subcutaneous layer of the surgical incision site. 1 ml of the mixture was injected proximal to the transverse wrist crease subcutaneously to release the distal part of the forearm deep fascia proximal to the carpal tunnel, as shown in Fig. 1. The hand and distal forearm were cleaned and draped just after finishing the injection, and CTR was performed directly without the need to wait extra time after testing the sensation at the incision area. If bilateral CTR was performed at the same setting, injection of both sides was performed first; then, the surgery was carried out in each hand sequentially.

**Fig. 2:**
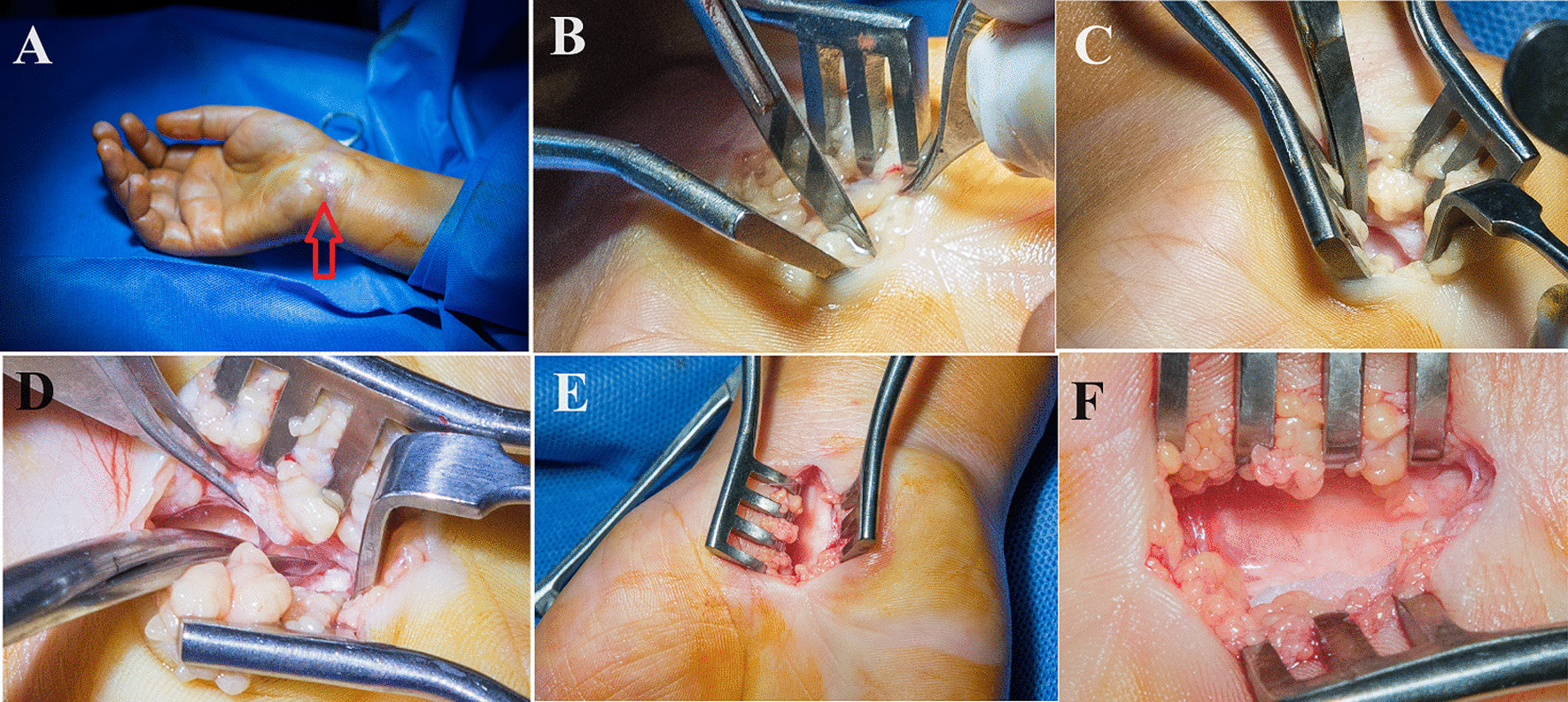
Carpal tunnel release under the mWALANT technique showing clear and bloodless field, **A** the injection site is evident by the slight swelling and skin elevation (red arrowhead), **B** subcutaneous dissection after skin incision. **C** releasing transverse carpal ligament distally, **D** releasing proximal part of transverse carpal ligament, **E** and **F** median nerve after complete release

All surgeries were performed in operative theater using magnifying surgical loupes by the same surgeon through a mini-open approach, as demonstrated in Fig. 2. Pain felt during the procedure was evaluated using the visual analog scale (VAS), and the patient was enquired when the pain was maximum.

### Postoperative and follow-up protocol

The procedure was performed as a daycare without hospital admission. Patients were discharged from the operative theater to the recovery area, where a well-trained nurse monitored their vital signs every 30 min for the first two hours, and the status of fingers vascularity was monitored by capillary refill time (should be < 2 s) and temperature changes which was checked by infrared surface thermometer.

Patients were discharged home, and no antibiotics were prescribed; however, on-demand analgesia was prescribed for two weeks (such as paracetamol or ibuprofen). Patients were instructed to attend the clinic immediately if they developed severe pain or a change in their fingers color. The patients were instructed to perform an active and passive full range of motion (ROM) of the fingers as tolerated to prevent adhesion formation and perform hand dry ADL.

The first follow-up visit was scheduled at one-week postoperatively for wound evaluation, changing the dressing, and ensuring that pain and numbness symptoms started to subside. Then, at two weeks postoperatively, the surgical wound was re-evaluated, and sutures were removed. From this moment on, patients were allowed to perform ADL as tolerated. Further, the following visits were scheduled at 2-, 6-, and 12-month postoperatively.

### Statistical analysis

A simple data description was provided as means and standard deviation (range) or frequencies and percentages calculated using the Microsoft Excel program.

## Results

### Patients and operative characteristics

The patients’ average age at surgery was 42.88 ± 13.03 years old (ranging from 15 to 83), and they were followed up for an average of 31 ± 17.17 months (ranging from 4 to 60).

In the 200 (100%) patients, both hands were affected in 108 (54%), the right side only in 55 (27.5%), and the left side only in 37 (18.5%) patients, giving a total number of 308 procedures. In all patients who were presented with bilateral affection, both hands were operated upon in the same setting except for two patients, where each side was operated upon in a different session. The average operative time was 9.5 ± 1.87 min (ranging from 7 to 13).

None (0.0%) of the patients needed top-up of local anesthesia or shift into general anesthesia, and no (0.0%) patients needed postoperative hospital stay or took time to recover from anesthesia.

### Outcomes and complications

The average VAS during the surgical procedure was 2.5 ± 2.1 (ranging from 1 to 4), mainly during infiltration of the local anesthesia (as stated by the patients); however, no patients reported discomfort during the surgical procedure itself. The average time to return to ADL for all patients was 6.9 ± 3.7 weeks (ranging from 2 to 17), where 180 (90%) patients reported a full return to their usual preoperative ADL within 1.5 months (average 4.7 ± 1.2 weeks (ranging from 2 to 6)). The remaining 10% who showed a delay in returning to ADL (complaining mainly of discomfort and sense of hand weakness) were scheduled for a rehabilitation program by the physiotherapist for strengthening hand grip, active and passive ROM of fingers, and pain relief. They showed improved grip strength, hand ROM and returned to ADL after an average of 11.3 ± 2.8 weeks (ranging from 7–17). Patients complained of postoperative pillar pain in 25 (8.1%) procedures. Those patients were reassured, and analgesia was prescribed; the pain was relieved after an average of 6.4 ± 2.1 months (ranging from 3 to 10). None (0.0%) of the patients showed recurrence of CTS symptoms by the last follow-up. No (0.0%) postoperative fingers ischemic or temperature changes in the operated hand. Two (1%) patients experienced an adrenaline rush in the form of tachycardia (average heart rate 112.5 ± 3.5 beats/min) that needed sedation and close monitoring by the anesthesiologist; they were discharged on the same day. One (0.5%) patient (who had uncontrolled diabetes mellitus) showed wound dehiscence and inflamed skin edges one week postoperatively; restriction from ADL, a broad-spectrum antibiotic, and daily dressing were prescribed. The wound completely healed at about the third week postoperatively.

## Discussion

CTS surgical release is one of the most common procedures performed, which evolved over the years from being an open procedure to being performed endoscopically [2, 4]. However, evolvement was not only in the surgical procedure itself but included introducing relatively newer anesthesia techniques, such as WALANT, which showed efficiency and wide adoption over the past few years [1, 6, 13, 16]. All the evolvement aimed at changing the procedure to become a daycare without the need for general anesthesia or hospital stay, which eventually will decrease the cost and help reduce the waiting lists and surgical caseload [16–20].

In the current study, although we used a relatively higher dose of epinephrine compared to what was described with the original WALANT technique, we reported no complications related to epinephrine injection; furthermore, no waiting time was needed for the drugs to start acting before proceeding with the surgical release.

To ensure proper vasoconstriction effect of the injected epinephrine before proceeding with surgical incision, the surgeon should wait for about 30 min, which was reported to be the most effective time (regarding bleeding control) in the study by Mckee et al. compared to the previously quoted seven minutes [14]. In the modification we proposed, we did not have to wait till the drugs were utterly working; furthermore, to ensure efficiency, we performed injection first, followed by draping (which usually took about 2–3 min before surgical incision); we believe that this step contributed to avoiding waiting for the reported 30 min before surgical incision.

The issue of time-saving was suggested as one of the advantages of the WALANT technique; this was confirmed even after endoscopic CTR, where Wellington et al. compared the results between WALANT (78 patients) versus sedation (53 patients) or local anesthesia with a tourniquet (25 patients); WALANT showed a significant reduction in the procedure time and total operative room time with no difference in the complications rate compared to the other procedures [18].

In the current series, the recorded pain had an average of 2.5 ± 2.1, mainly reported by patients at the start of the injection process; however, no pain was reported during the main surgical procedure. This discomfort or pain varied among previous studies; Ralte et al. compared the results of using local anesthesia with a tourniquet and WALANT technique during CTR; they reported equal discomfort at the time of infiltration in both groups, but the group in which tourniquet was used, patients reported discomfort during tourniquet release [21]. In a study by Faraz et al., including 18 patients who underwent CTR utilizing the WALANT technique, they reported lower pain levels perceived by the patients (as measured by VAS), both during the procedure and two weeks postoperatively, 3.1 ± 1.2 and 1.67 ± 0.9, respectively [17]. On the contrary, in a study comparing pain after open CTR, Tulipan et al. reported postoperative higher VAS scores in the 81 patients operated under the WALANT technique (VAS was 2.3) compared to 149 patients operated under sedation (VAS was 1.8); however, this difference disappeared after three months, and their patients showed no difference regarding satisfaction with either procedure [22].

One of the concerns while adopting the WALANT technique is the possibility of epinephrine-related complications [13], which could range from minor adverse effects, such as symptoms related to adrenaline rush or vasovagal symptoms, to serious adverse effects, including cardiac arrhythmias [23]. In the current study, we monitored these adverse effects carefully, especially since we used undiluted epinephrine; we only had symptoms of adrenaline rush in 1% of the patients who were treated efficiently without further complications or need for hospitalization. Although rarely reported, digit ischemia is a serious possible risk associated with epinephrine injection [13, 24, 25]; however, we did not encounter such a complication in the current series.

It is worth noting that although the WALANT technique is widely adopted for various surgical procedures (including CTS and flexor tendon injury repair), some authors reported a lack of evidence to prove its superior advantages and safety compared to other techniques (such as general anesthesia, sedation, and regional anesthesia), particularly concerning intraoperative and postoperative complications [20, 26, 27]. Furthermore, in a literature review by Fulchignoni et al., the authors concluded no difference in the outcomes after flexor tendon injuries surgical repair after WALANT compared to the traditional anesthesia techniques [20].

The current study had some limitations. First, the noncomparative nature of the study stood against proving the superiority or inferiority of the mWALANT compared to the original WALANT technique. Second, we did not calculate the exact time needed to perform the surgical incision, as we did not have to wait, and the procedure was carried out directly after finishing the draping. Last, we did not report on the cost-effectiveness and time savings of the mWALANT; however, this was provided for the original WALANT technique in many reports [1, 13, 17, 28].

## Conclusions

Using undiluted epinephrine during the mWALANT technique is safe and effective, with no need to wait until the drugs fully function, and no epinephrine-related complications were encountered apart from occasional adrenaline rush symptoms. However, further comparative studies are mandatory to determine the time needed to obtain the proper vasoconstriction effect between the original WALANT and the mWALANT techniques; furthermore, comparing the cost-effectiveness of both procedures is paramount.

## Data Availability

All the data related to the study are mentioned within the manuscript; however, the raw data are available with the corresponding author and will be provided upon a written request.

## Abbreviations

CTS: Carpal tunnel syndrome
ADL: Activities of daily living
CTR: Carpal tunnel release
WALANT: Wide-awake local anesthesia no tourniquet
mWALANT: Modified WALANT
APB: Abductor pollicis Brevis
VAS: Visual analog scale
